# The impact of CD3ζ ITAM multiplicity and sequence on CAR T-cell survival and function

**DOI:** 10.3389/fimmu.2024.1509980

**Published:** 2025-01-16

**Authors:** Shubhabrata Majumdar, Hilda Echelibe, Maria Bettini, Matthew L. Bettini

**Affiliations:** ^1^ Immunology Graduate Program, Baylor College of Medicine, Houston, TX, United States; ^2^ Department of Pathology, University of Utah, Salt Lake City, UT, United States

**Keywords:** chimeric antigen receptor (CAR), CD3, immunoreceptor tyrosine-based activation motif (ITAM), signaling, cytokines

## Abstract

**Introduction:**

Chimeric antigen receptor (CAR) expressing T-cells have shown great promise for the future of cancer immunotherapy with the recent clinical successes achieved in treating different hematologic cancers. Despite these early successes, several challenges remain in the field that require to be solved for the therapy to be more efficacious. One such challenge is the lack of long-term persistence of CD28 based CAR T-cells in patients. Although, CD28 based CAR T-cells elicit a robust acute anti-tumor response, they are more prone to early exhaustion, terminal differentiation and cell death due to their strong signaling patterns. Hence attenuation of signaling strength in CD28 based CARs is an accepted strategy to improve long-term CAR T-cell function and persistence in patients. Previous studies with the conventional T-cell receptor (TCR) have suggested that manipulation of CD3 immunoreceptor tyrosine-based activation motif (ITAM) sequences can alter TCR signaling strength. Based on these studies, we have designed 2^nd^ generation murine anti-CD19 CD28 based CARs with restricted CD3ζ ITAM sequence diversity while maintaining a multiplicity of three. They are called ζAAA, ζBBB and ζCCC based on which CD3ζ ITAM they express. The goal of the study is to understand the non-redundant signaling properties of the individual CD3ζ ITAMs and their effect on CAR T-cell function. We hypothesized that the individual CD3ζ ITAMs will exhibit unique signaling properties in the ITAM restricted CARs which may allow for optimization of CAR signaling and improve CAR T-cell persistence and function.

**Method:**

We subjected the ITAM restricted CAR T cells to various conditions of in vitro stimulation using CD19+ tumor cells or CD19-coated magnetic beads. Immunoblotting and flow cytometry based Ca2+ signaling assays were used to quantify signaling differences. Functional differences were studied using in vitro cytotoxicity, degranulation and cytokine expression assays. CAR T cell exhaustion and differentiation were studied using an in vitro exhaustion assay.

**Results:**

We observed that ζAAA CARs had stronger signaling strength compared to ζBBB and ζCCC CARs. The signaling differences were reflected in their functional activation profiles with T-cells expressing ζAAA CARs having a strong activation profile and ζCCC CARs having a weak activation profile. ζCCC CAR T cells were less prone to differentiation and exhaustion.

**Discussion:**

Since, weaker signaling ζCCC CARs favored less cell death, exhaustion and differentiation, they might be better candidates for improving long term survival and persistence of CAR T cells in patients.

## 1 Introduction

CARs are artificial receptors that have been designed to recognize user defined antigens in an MHC independent manner and activate downstream signaling pathways that lead to T-cell activation in response to antigen recognition. CAR T-cells have revolutionized the field of cancer immunotherapy with the successful clinical application in the treatment of several refractory B-malignancies like leukemias, lymphomas and multiple myeloma (1–5). Current FDA approved CARs consist of an extra-cellular antigen specific antibody derived single-chain variable fragment (scFv), a transmembrane domain, a co-stimulatory domain (CD28 or 41BB) and typically the CD3ζ chain (6–8). The CD3ζ chain is phosphorylated at specific conserved tyrosine residues located in signaling motifs called ITAMs to initiate CAR signaling (8–12). Despite the early clinical successes, there are several challenges to overcome and further mechanistic studies are required to increase efficacy and the range of cancer treatment.

One of the major challenges facing CAR T-cell therapy is a lack of long-term persistence of CAR T-cells in patients. This is especially true for CD28 based CARs which have been shown to elicit a robust acute anti-tumor response because of stronger signaling strength and faster kinetics (6, 12). However, similar to the studies with the conventional TCR impact on T-cell function, stronger CAR signaling leads to poor persistence because of more activation induced cell death, early T-cell exhaustion and terminal differentiation (13–15). Hence, recent strategies have been focused on the manipulation of the intra-cellular signaling domains of CARs to attenuate signaling strength and improve persistence.

One strategy is to reduce the number of ITAMs in the CD3ζ chain where CAR signaling is initiated (16, 17). This is based on prior observations with the conventional TCR, where reducing functional ITAM numbers is associated with attenuation of signaling strength and subsequent impact on T cell function (9, 18, 19). A recent study demonstrated that the single ITAM CAR 1928ζ1XX had better persistence and tumor killing efficacy with more memory-like CAR T-cells (16). However, reduction in ITAM number might come at a cost of reduced sensitivity to low antigen density tumors and allow tumor antigen escape (20). Prior studies with the conventional TCR have also suggested that despite the quantitative significance of ITAM number, there might be qualitative/kinetic differences in the individual ITAM signaling properties based on the amino acid differences surrounding the conserved tyrosine residues (19, 21). One such study showed that restricting ITAM diversity to single ITAM sequences while maintaining ITAM multiplicity changes TCR signaling strength (21). Additionally, artificial membrane/liposome based, and computational studies have suggested that there might be inherent differences in the kinetics of tyrosine phosphorylation based on the ITAM sequence. In this manuscript, we address whether individual CD3ζ ITAMs A, B, C in CARs differ in their signaling and its effect on CAR T-cell function and persistence.

In this study we have generated murine 19CD28ζ CARs with restricted CD3ζ ITAM diversity. Instead of expressing the three different CD3ζ ITAMs A, B and C as in the conventional CARs (ζABC), our modified CARs express three copies of either A (ζAAA), B (ζBBB) or C (ζCCC). We hypothesized that the individual CD3ζ ITAMs will exhibit non-redundant signaling properties in the diversity restricted CARs which may allow for optimization of CAR signaling and improve CAR T-cell persistence and function. Our observations suggest that the individual CD3ζ ITAMs confer differential signaling strength to the CARs. The weaker signaling CAR T-cells also have a weaker activation profile. Additionally, the weaker signaling ζCCC are less prone to exhaustion and are less differentiated which is indicative of longer persistence. Overall, this study sheds light on the individual signaling tendencies of CD3ζ ITAMs and provides further opportunities for the manipulation of CAR design to reduce CAR T-cell exhaustion and enhance persistence.

## 2 Results

### 2.1 Construction of ITAM restricted murine anti-CD19 CARs

**Figure 1:**
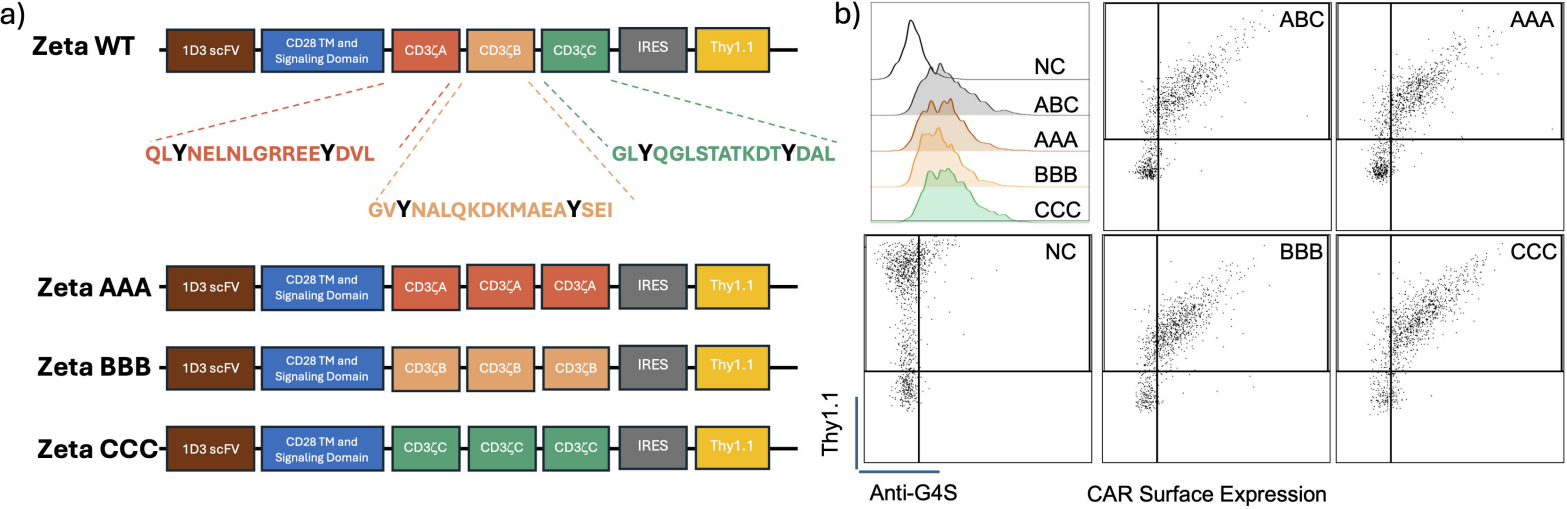
Construct design for diversity restricted ITAM CARs and their expression in primary CD8 T-cells. **(A)** Schematic representation of the construct design for the ITAM restricted CARs along with the individual amino acid sequences of the individual ITAMs. **(B)** Flow plots showing CD8+ T-Cell CAR expression in positive correlation with reporter Thy1.1 expression. CAR expression detected using an antibody targeting the conserved G4S sequence.

To test our hypothesis, we generated ITAM restricted murine anti-CD19 CD28 CARs that express three copies of a single CD3ζ ITAM instead of the three different CD3ζ ITAMs as in the conventional CAR design. The CAR backbone was previously published and obtained from Addgene (Plasmid #107226) (17). It consists of the 1D3 clone anti-CD19 binding scFv domain, a CD28 transmembrane and signaling domain, followed by the CD3ζ ITAMs (**Figure 1A**). The newly designed CARs are designated as ζAAA, ζBBB and ζCCC depending on which CD3ζ ITAM sequence is used. Since we want to compare the signaling properties of the ITAM restricted CARs, we wanted to first verify the CAR surface expression levels were similar. We tested each construct for cell surface expression by retrovirally transducing primary mouse CD8+ T-cells and staining with an antibody specific for the linker sequence in the CAR construct. Using the anti-G4S antibody, we observed that the CARs are expressed on the cell surface at similar levels, making signaling studies with these CARs possible (**Figure 1B**).

### 2.2 ITAM restricted CARs differ in their signaling properties

**Figure 2:**
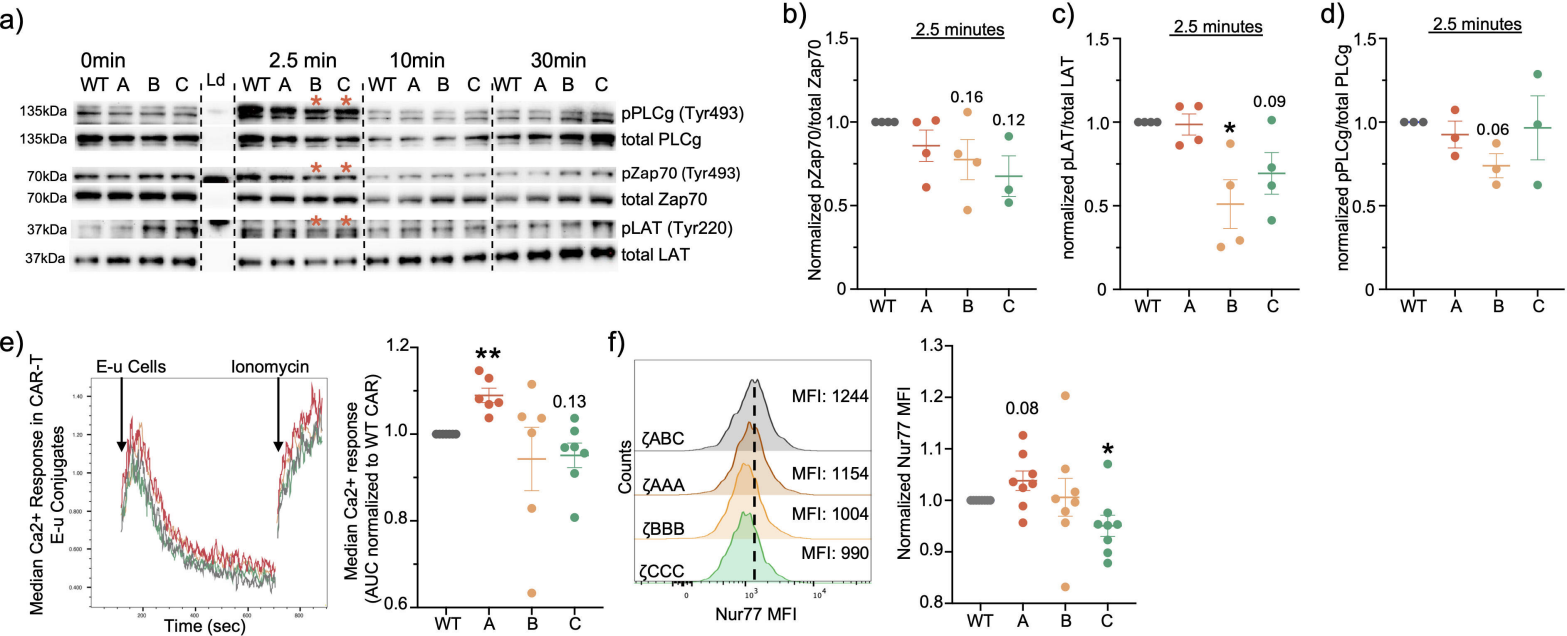
ITAM restricted CARs exhibit differential signaling. **(A)** Representative immunoblots of CAR proximal signaling kinetics from whole lysate of CD8+ CAR-T cells stimulated with CD19+ dynabeads. **(B-D)** Relative quantification of proximal signaling molecules at peak signaling time-point of 2.5min. **(E)** Representative ratiometric Ca2+ response curve in Indo-1 labelled CD8+ CAR T-cells stimulated with CD19+ E-u cells and its relative quantification of median Ca2+ response in CD8+ CAR-T cells as area under the curve. **(F)** Nur77 expression quantified in CD8+ CAR-T cells stimulated with CD19+ E-u cells for 6hrs at 1:2 E:T ratio. Each dot represents an individual mouse donor. Normalized data was analyzed using one-sample T-test. Red asterisk designates 2.5 min timepoint for **(B, C)**. Significance representation *p<0.05, **p<0.01.

To compare the signaling properties of the individual CD3ζ ITAMs, we stimulated our CAR T-cells *in vitro* using CD19 coated dyna-beads for different time-points and quantified the signaling differences using immunoblotting. We measured phosphorylation of proximal protein targets that have been previously shown to be activated downstream of TCR signaling. We first observed that the signaling kinetics are similar to that of the TCR with proximal signaling peaking at the 2.5min timepoint (**Figure 2A**; **Supplementary Figures 1A-C**). However, at the peak proximal signaling timepoint (2.5min), we observed that the proximal signaling molecule LAT was less phosphorylated in ζBBB, while for Zap70, ζBBB and ζCCC CARs were also slightly decreased in comparison to the ζABC CAR, suggesting weaker activation (**Figures 2B-D**). To complement this observation, we tested the Ca2+ signaling response in each of the CAR-T cell constructs, which is initiated downstream of PLCγ activation. Ca2+ sensitive Indo1-AM labelled CAR-T cells were stimulated with CD19+ E-µ ALL cells and Ca2+ response kinetics was measured using flow cytometry (22). We observed that ζAAA CARs had a significantly greater median Ca2+ response compared to ζABC CARs, while ζCCC trended towards a lower response compared to ζABC CARs (**Figure 2E**). To assess the impact of ITAM restricted CARs on downstream transcriptional activity, we measured the expression of Ca2+ responsive transcription factor Nur77 (23, 24). In concordance with the Ca2+ response, we observed ζCCC CARs to have a significantly lower expression of Nur77 while ζAAA was slightly higher compared to the wild type ITAM CAR (**Figure 2F**). Together, these data indicate each ITAM sequence allows for differential CD19-CAR intracellular signaling, therefore we next wanted to test whether the alteration in signal transduction translated into altered functional outcomes.

### 2.3 ITAM restricted CARs are functionally unique *in vitro*

**Figure 3:**
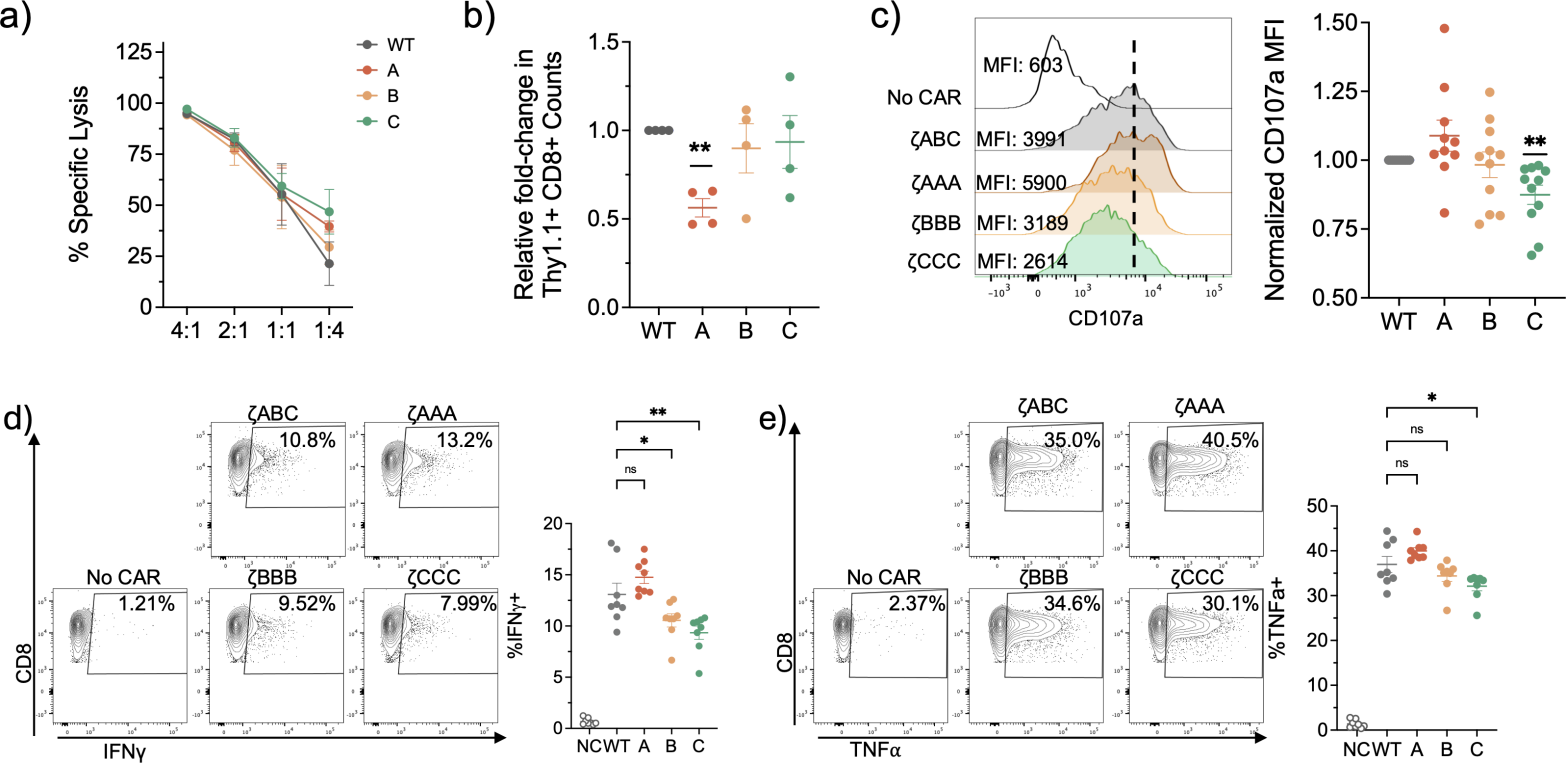
*In vitro* functional characterization of ITAM restricted CAR-T cells suggest Zeta B and Zeta C CARs have weaker functional activation profile upon antigen encounter. **(A)** Representative Specific lysis of CD19+ E-u tumor cells in a 24-hour dye release co-culture assay. n=3. **(B)** CAR-T cell survival 24 hours post-killing in 1:2 E:T ratio co-culture with CD19+ E-u tumor cells. Data pooled from 2 independent experiments. **(C)** CD107a degranulation assay with CD8+ CAR-T cells co-cultured with CD19+ E-u cells at a 1:2 E:T ratio for 4hrs. Representative (left) and normalized to WT group (right). Data pooled from 4 independent experiments. **(D, E)** Cytokine activation profile of CD8+ CAR-T cells stimulated with CD19+ E-u cells in 1:2 E:T ratio for 6hrs in presence of BFA and monensin. Data pooled from 3 independent experiments. Each dot is representative of an individual mouse donor. Normalized data analyzed using one sample T-test. Cytokine expression percentages analyzed using one-way ANOVA followed by a *post-hoc* Dunnett’s test. Significance representation *p<0.05, **p<0.01; ns, not significant.

We next performed *in vitro* functional assays to assess the effect of the observed signaling differences on CAR-T function. We first performed a dye-release cytotoxicity assay where we co-cultured our CAR-T cells with dye-labelled CD19+ E-µ tumor cells at different E:T ratios for 16 hours. We observed all our CAR T-cells were similarly effective in their specific cytotoxicity (**Figure 3A**). Interestingly, when we quantified the viable CD8+ CAR T-cell counts, we observed ζAAA CARs had reduced cell numbers compared to the ζABC CARs which suggests increased exhaustion or AICD (**Figure 3B**). We next performed a CD107a degranulation assay to assess lytic effector function. Each ITAM restrict CAR T cell was co-cultured with the CD19+ E-µ cells in a 1:1 E:T ratio for 6 hours in the presence of PE-conjugated CD107a antibody. Here we observed ζAAA and ζBBB CAR T-cells were similar to ζABC in terms of degranulation (**Figure 3C**). However, ζCCC CAR T cells demonstrated less staining of surface CD107a, suggesting they are less likely to degranulate upon activation (**Figure 3C**). Next, we performed an *in vitro* cytokine expression assay to measure effector cytokine expression of IFNγ, TNFα and IL2. CAR-T cells were co-cultured with CD19+ E-µ cells for 6 hours in the presence of Brefeldin A and monensin and expression of IFNγ, TNFα and IL2 was measured by intracellular staining. Similar to CD107a expression, we observed ζCCC CAR T-cells expressed less IFNγ and TNFα (**Figures 3D, E**). However, ζBBB showed a selective decrease in IFNγ expression while maintaining similar levels of TNFα and IL2 expression compared to ζABC (**Figures 3D, E**; **Supplementary Figure 2**). Overall, these data suggest that ζCCC CAR T-cells have a weaker activation profile upon encountering tumor antigen that may lead to less effector function but better survival over time.

### 2.4 ζBBB CAR T-cells are more prone to exhaustion under conditions of chronic stimulation

**Figure 4:**
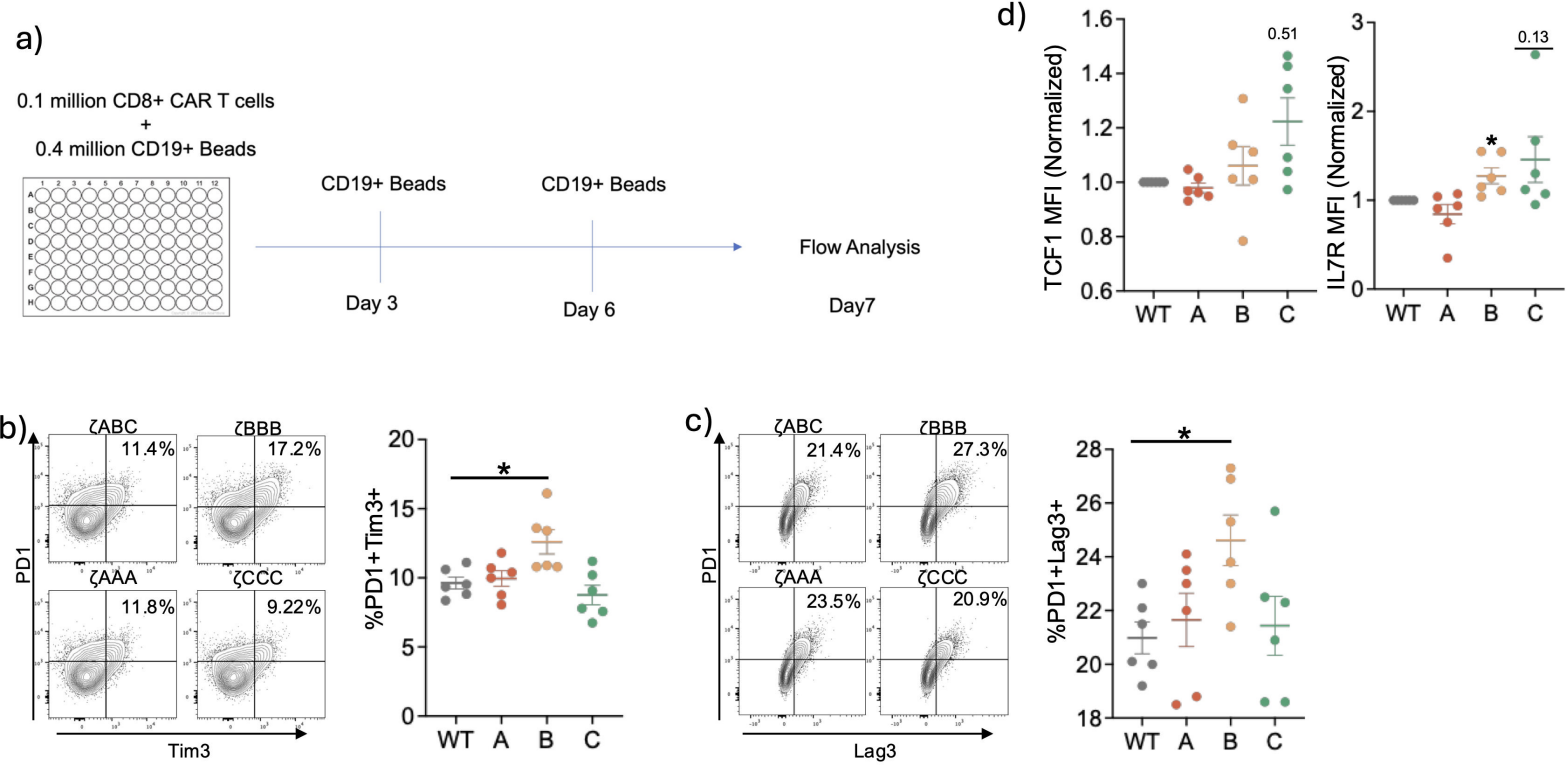
Long-term *in vitro* stimulation of ITAM restricted CARs suggest Zeta B are more prone to exhaustion and Zeta C CARs are less prone to exhaustion. **(A)** Experimental setup for *in vitro* exhaustion assay. CD8+ CAR-T cells were stimulated with CD19 beads in the presence of IL-2 for 7 days. Stimulations were repeated every 3 days and cells were analyzed on Day 7. **(B, C)** Representative flow plots and combined data of CD8+ CAR T-cells expressing inhibitory receptors. **(D)** Relative expression levels of TCF1 and IL7R in CD8+ CAR-T cells at Day7. Data pooled from two independent experiments. Each dot represents an individual mouse donor. Normalized data analyzed using one sample T-test. Percentages were analyzed using one-way ANOVA with *post-hoc* Dunnett’s test. Significance representation *p<0.05.

Since previous studies with the conventional T cell have suggested that weaker activation favors less exhaustion, we tested our ITAM restricted CAR T-cellsfor signs of exhaustion in an *in vitro* exhaustion assay (25–27). We subjected the CAR T-cells to chronic stimulation conditions by culturing them in the presence of CD19+ dyna-beads for 7 days in the presence of IL-2 and analyzed them for expression of exhaustion related inhibitory receptors (**Figure 4A**). After repeated stimulation, we observed ζCCC CAR T-cells had slightly lower co-expression of inhibitory receptors PD1 and Tim3, whereas ζBBB had significantly higher expression compared to ζABC (**Figures 4B, C**, **Supplementary Figures 3A–C**). Interestingly, ζCCC also exhibited higher expression of TCF1 and IL7R, both of which are associated with less differentiated more memory-like cells (**Figure 4D**). Together, these data indicate ITAM sequence can lead to differential signal transduction and functional outcomes.

## 3 Discussion

In this study, we aimed to test the signaling properties of the individual CD3ζ ITAMs in the context of CD28-based CARs to better understand the impact of ITAM sequence manipulation to improve CAR T cell function and persistence through reduction of terminal differentiation, exhaustion and activation induced cell death (16, 20).

**Figure 5:**
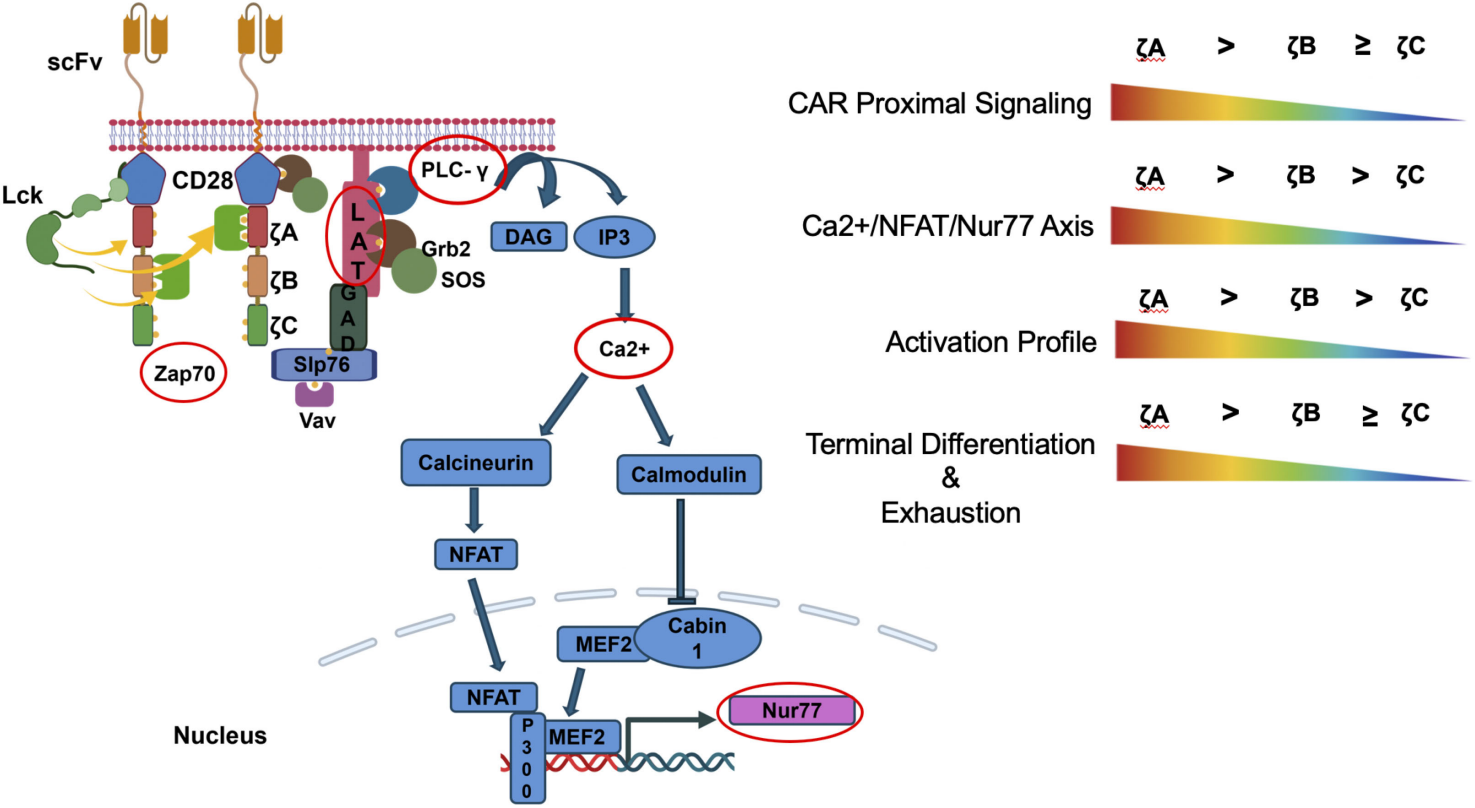
Individual CD3ζ ITAMs differ in their signaling properties. Overall ζCCC CARs have weaker signaling and activation profile while ζAAA CARs had a stronger signaling and activation profile. The weaker signaling ζCCC CARs also showed less terminal differentiation and were less prone to exhaustion under conditions of chronic stimulation.

As per our hypothesis, we observed that the ITAM restricted CAR T-cells had unique signaling profiles upon stimulation with CD19+ beads or tumor cells (**Figure 5**). Our results suggest that the ζBBB and ζCCC CAR T-cells have weaker phosphorylation/activation of ITAM proximal signaling molecules like Zap70, LAT and PLCγ while ζAAA CAR T-cells have comparable levels of activation to that of the ζABC CAR T-cells. However, when we measured Ca2+ signaling response, a pathway activated downstream of PLCγ signaling, we observed ζAAA CAR T-cells have higher levels of Ca2+ signaling while ζCCC CAR T-cells have a trending lower Ca2+ response. Importantly, this effect was reflected at the downstream gene expression level of Nur77, which is a transcription factor whose transcription is regulated by Ca2+ dependent NFAT1-MEF2 co-transcriptional activity (23, 24, 28). To this end, we observed that ζCCC CAR T-cells had a lower Nur77 expression while ζAAA CAR T-cells had a trending higher expression in comparison to ζABC CARs, after CD19+ tumor cell stimulation. These observations suggested that ζAAA CAR T-cells have an overall stronger signaling strength, while ζCCC CAR T-cells have weaker signaling strength. Although we show differential phosphorylation of downstream signaling targets, further investigation is required to better undertand how the amino acid sequences surrounding the conserved tyrosine residues lead to the observed differences. We hypothesize that the observed differences are a consequence of preferential binding and differential phosphorylation/dephosphorylation kinetics of the conserved tyrosine residues by the Src family tyrosine kinases Lck/Fyn and the tyrosine phosphatase CD45 (11, 28, 29). This would lead to different rates of Zap70 recruitment and activation of downstream pathways. In this regard, our results are in accordance with a previous study, where phosphorylation levels of CD3ζ ITAM A were observed to be higher than ITAM B and ITAM C by mass spectrometry in Jurkat T-cells activated using anti-CD3 stimulation. Using an on-membrane FRET system, the authors suggested this is a consequence of less efficient dephosphorylation of ITAM A by CD45 (30). There is also the possibility of Zap70 binding to the different SH2 domain binding sites at different rates. This is supported by early studies using synthetic phospho-peptide binding methods, which suggest that Zap70 has a higher binding affinity for doubly phosphorylated ITAM A than ITAMs B and C, with some ambiguity in the hierarchy between ITAMs B and C (31–33). It will be of importance to investigate the signalosome of the CAR in future studies to better elucidate the relative protein interactions and binding kinetics of the zeta chain ITAMs. Although we have focused only on the quantitative differences in CD3ζ ITAM signaling in this study, future studies will test whether there are any qualitative differences in recruitment of proximal signaling molecules.

In parallel, we observed the unique signaling profiles led to differential T cell functional responses with each ITAM construct. We observed ζCCC CARs had a lower activation profile overall, while ζAAA CAR T-cells had a stronger activation profile in terms of the rate of degranulation and expression of effector cytokines IFNγ and TNFα. Although we did not detect any difference in specific cytotoxicity, we did observe less ζAAA CAR T-cells compared to other CAR T-cells after tumor killing. This might suggest that ζAAA CAR T-cells are more prone to activation induced cell death as a result of higher signaling. Additionally, similar to previous studies indicating conventional TCRs with weaker signaling strength favors less T-cell exhaustion, the ζCCC CAR-T cells had lower expression of inhibitory receptors that are associated with exhausted T-cells compared to ζABC CAR-T cells which may be due to a decrease in Nur77 signaling pathway.

Overall, our results suggest modifying ITAM sequence can regulate CAR signaling strength without having to reduce ITAM multiplicity. Based on our findings we propose that ζCCC CARs will perform better *in vivo* as they have a weaker activation profile and are less prone to exhaustion.

## 4 Materials and methods

### 4.1 Design and generation of CAR constructs

MSGV-1D3-28ζ All ITAMs intact was a gift from James Kochenderfer & Steven Rosenberg (Addgene plasmid # 107226; http://n2t.net/addgene:107226; RRID: Addgene_107226).

### 4.2 Production of retroviral supernatant

Platinum-E (Plat-E) cells obtained from ATCC were used as transient retroviral producers. In brief, 1 million Plat-E cells were plated in 10% complete DMEM. The following day, Plat-E cells were transfected with 6µg vector plasmid using TransIT-LT1 (Mirus) transfection reagent in the presence of 25uM chloroquine. The media was replaced 6 hours later and collected at 48- and 72-hour timepoints for transduction.

### 4.3 T-cell isolation, transduction and expansion

On Day 0, T-cells were isolated from spleen and lymph nodes (inguinal, axillary and cervical) of 8–12-week-old donor B6 mice. Single-cell suspensions from the organs were subjected to RBC lysis followed by staining with anti-CD8 and/or anti-CD4 biotinylated antibodies. Miltenyi Streptavidin magnetic beads were used to magnetically enrich CD8+ and/or CD4+ T-cells. T-cells were activated in 24-well plates coated with 10ug/ml anti-CD3 and 2ug/ml anti-CD28 at a density of ~0.5 million cells per well. T-cells were stimulated for 24 hours before transduction.

On Day 1, activated T-cells were transduced using retroviral supernatant obtained from the Plat-E cells. Briefly, the cellular debris was removed from the Plat-E supernatant by centrifuging at 300g for 5 minutes. Polybrene (6ug/ml) and hIL-2 (50U/ml) were added to the supernatants and cells were spin-transduced at 1300g for 1 hour at 37°C. After transduction, T-cells were allowed to rest in the viral supernatant for 1 hour in the 37°C CO2 incubator before replacing media with 10% complete RPMI containing 50U/ml hIL-2. The process was repeated 24 hours later. The transduction efficiency was in the range of 30-50%.

After 48-72 hours of activation, on Day 3 the T-cells were removed from the anti-CD3 and anti-CD28 coated plates and transferred to T-cell expansion medium containing 10% complete RPMI supplemented with 10ng/ml hIL-7 and 10ng/ml hIL-15. T-cells were maintained at density of ~0.5 million cells/ml. On Day 5, CAR expressing T-cells were magnetically enriched using anti-Thy1.1 biotinylated antibodies and streptavidin magnetic beads. Post-enrichment CAR T cell populations were ~90% pure. For *in vitro* experiments, Day 7 - Day 10 cells were used.

### 4.4 Tumor cell line and culture conditions

CD19+ E-µ ALL tumor cell line was obtained from Dr. Marco L. Davila’s lab. Since E-µ cells cannot expand on their own, they are grown as a co-culture with NIH/3T3 cells. 3T3 cells were cultured in 5% DMEM with ciprofloxacin. One day prior to plating the E-µ cells, 3T3 cells were X-ray irradiated at 30Gy and plated at 1 million cells per 10cm plate. Next day, E-µ cells are added and expanded in a 50/50 mix of 10% complete RPMI and 10% complete IMDM.

### 4.5 Preparation of CD19+ magnetic dyna-beads

M-450 tosylactivated dyna-beads (Invitrogen) were washed and resuspended in 0.1M sodium phosphate buffer (pH 7.8) at 4x10^8^ beads/ml and incubated overnight with 150ug of recombinant murine CD19 (R&D Systems) with gentle rotation. The beads were then washed 3 times in wash buffer (1X PBS with 0.1% BSA and 2mM EDTA pH 7.4) at 4°C for 5 min each with constant mixing. After washing, the beads were stored at 4°C in storage buffer (1X PBS with 0.02% sodium azide, 0.1% BSA and 2mM EDTA). Before use, beads were washed 3 times in complete RPMI at 4°C with constant mixing.

### 4.6 Immunoblotting and quantification

CAR T-cells were counted and rested in 1X PBS with calcium and magnesium for 30min at room temperature. Two million CD8+ CAR T-cells were stimulated with 6 million CD19+ dynabeads by spinning down for 1min and incubating at 37°C for the mentioned timepoints. Signaling was halted by transferring cells to ice and immediately lysing with 1% NP-40 in deionized water containing Halt Protease/Phosphatase inhibitor cocktail. Lysis was performed for 30min at 4°C with constant mixing. After spinning down debris, equal volumes of protein lysate were mixed with Laemmli’s buffer (Bio-Rad) with 2-mercaptoethanol and denatured at 95°C for 5minutes. Samples were then run in pre-cast gels (Invitrogen, NuPage Bis-Tris midi protein gels, 4-12%, 1.0mm) in MOPS SDS running buffer (Invitrogen). Immunoblotting was performed using Bio-rad’s Trans-blot Turbo semi-dry transfer machine onto a PVDF membrane. The membrane was blocked for 1 hour at room temperature using 3% BSA in tris buffered saline with 0.1% Tween20 (0.1% TBS-T). Primary antibody staining was performed overnight at 4°C in 3% BSA 0.1%TBS-T. Secondary antibody staining was performed at room temperature for 1 hour in 3% BSA 0.1%TBS-T. In between and after staining, the membrane was washed with 0.1% TBS-T 3X for 5min each. Bands were detected using chemiluminescence. Densitometric analysis was performed using Image Lab 6.1. Protein phosphorylation was quantified as a ratio of phospho band intensity and total protein band intensity. Ration was then expressed as fold change relative to unstimulated cells.

### 4.7 Ca^2+^ signaling assay and quantification

CD8+ CAR T-cells were labelled with the Ca^2+^ sensitive ratiometric Indo-1AM dye for detection of Ca^2+^ signaling. Briefly, the CAR-T cells were washed in 1X PBS and incubated with 5uM Indo-1 AM dye in PBS for 30min in 37°C incubator. After incubation, the cells were washed twice and resuspended in Ca2+ signaling buffer (Hank’s Balanced Salt Solution Cat# 55037C with calcium, magnesium and no phenol red, plus 10% FCS and 25mM HEPES). CAR-T cells were then stained for Thy1.1 for 10 minutes at room temperature. After washing and prior to flow cytometric analysis, the CAR T-cells were stored on ice in 5ml flow tubes at 1 million cells/500ul. The E-µ cells were labelled with 5uM Cell Proliferation Dye eFluor™ 670 (eBioscience™) in 1X PBS like the CAR-T cells and stored on ice at 2 million cells/500ul. Before running each sample on the flow cytometer, the cells were warmed in a 37°C water-bath for 15minutes.

Before adding the E-µ cells, the CAR-T cells were run on flow cytometer for 1min to collect baseline Ca^2+^ bound/Ca^2+^ free ratio. Flow rate is maintained constant at ~1000 events/sec across samples. At 1 min, the tubes were taken off with recording still ongoing, and 2 million E-u cells were added, mixed at allowed to form T:E conjugates by centrifugation at 300g for 25sec. At the 2 min timepoint, the tube was put back into run mode and data was recorded for 10 minutes to capture Ca^2+^ response. After 10 minutes, the tube was taken off the cytometer and ionomycin (~1ug/ml) was added to check maximum Ca^2+^ response.

For measurement of Ca^2+^ response, we gated on Thy1.1+ ef647+ conjugates and created a kinetics curve for median of *Ca^2+^ bound/Ca^2+^ free ratio* derived parameter. Gates were set for the peak response (120sec to 500sec) and area under curve was calculated and plotted relative to the ζABC CAR.

### 4.8 Dye release specific cytotoxicity assay

E-µ cells were labelled with 5uM eFluor™ 670 dye in 1X PBS. Thy1.1+ CAR T-cells were co-cultured with 50,000 dye labelled E-µ cells at the mentioned E:T ratios in a 96-well flat-bottomed plate for ~15 hours at 37°C. Dye+ E-µ cell numbers were quantified using flow cytometry and %specific lysis was calculated as


$$\begin{array}{c} \%\;\text{Specific}\;\text{Lysis}\\ =\frac{\left(Control\;ef647+Counts\;-\;CAR\;ef647+\;Counts\right)}{Control\;ef647+Counts}\;\times 100 \end{array}$$

### 4.9 *In vitro* cytokine stimulation assay

A total of 100,000 Thy1.1+ CAR T-cells were co-cultured with 200,000 E-µ cells in 10% complete RPMI at 37°C for 6 hours in 96 well round-bottomed plates. Brefeldin A (5ug/ml) and Monensin (2uM) were added at the beginning of incubation to prevent secretion of cytokines. Cytokine expression was analyzed by intra-cellular staining and flow cytometry.

### 4.10 Degranulation assay

A total of 100,000 Thy1.1+ CAR T-cells were co-cultured with 200,000 E-µ cells in 10% RPMI at 37°C in 96-well flat-bottom plates. Phycoerythrin (PE) labelled anti-CD107a (LAMP-1) antibody was added to each well at 1ug/ml final concentration at the beginning of the incubation to label CD107a exposed on the cell membrane as a result of T cell degranulation.

### 4.11 *In vitro* exhaustion assay

A total of 100,000 Thy1.1+ CD8+ CAR T-cells (Day 7 post-activation) were incubated with 400,000 CD19 coated dyna-beads on Day 0 in flat-bottomed 96-well plates in 10% complete RPMI containing 50U/ml IL-2. CAR-T cells were re-stimulated with 400,000 CD19 coated beads at Day 3 and Day 6. Media was replaced every 1.5 days with fresh RPMI containing 50U/ml IL-2. On Day 7, beads were removed, and cells were analyzed by flow cytometry.

### 4.12 Flow cytometry

Single-cell suspensions were stained in buffer containing PBS, 3% v/v FCS and 0.05% w/v sodium azide. Surface staining was performed on ice for 20 minutes after Fc receptor blocking with FcBlock (Biolegend) for 10 minutes. Live/Dead staining was performed using Zombie Red (Biolegend) in 1X PBS for 30 min on ice. For intra-cellular staining, cells were fixed with FoxP3 Fix/Perm reagent (eBioscience) and then stained under permeabilizing conditions overnight at 4°C in Permeabilization Buffer (eBioscience). Flow cytometry data were acquired on a BD LSRFortessa (Becton Dickinson) flow cytometer and analyzed using the FlowJo 10 software (FlowJo LLC).

## Data Availability

The original contributions presented in the study are included in the article/**Supplementary Material**. Further inquiries can be directed to the corresponding author.
