# Supplementary material for: The impact of CD3ζ ITAM multiplicity and sequence on CAR T-cell survival and function
Source: Front Immunol. 2025 Jan 16;15:1509980. doi: 10.3389/fimmu.2024.1509980 (PMC11779709; doi:10.3389/fimmu.2024.1509980)
Supplement: Supplementary file 1 [file DataSheet1.pdf]

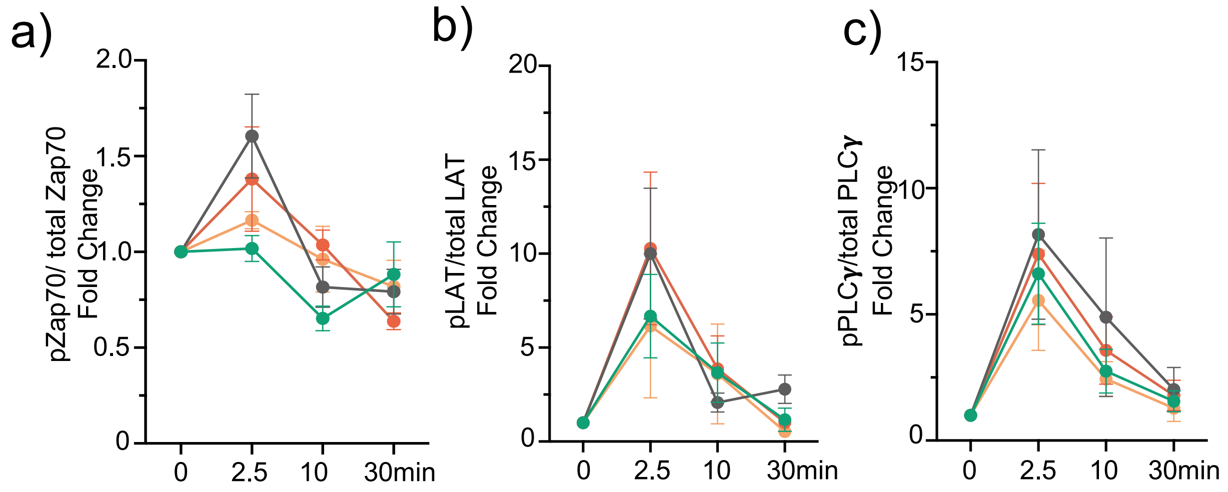

Supplemental Figure 1: ITAM restricted CARs exhibit differential signaling. A-c) Fold change time course of CAR proximal signaling kinetics from whole lysate of CD8+ CAR-T cells stimulated with CD19+ dynabeads.

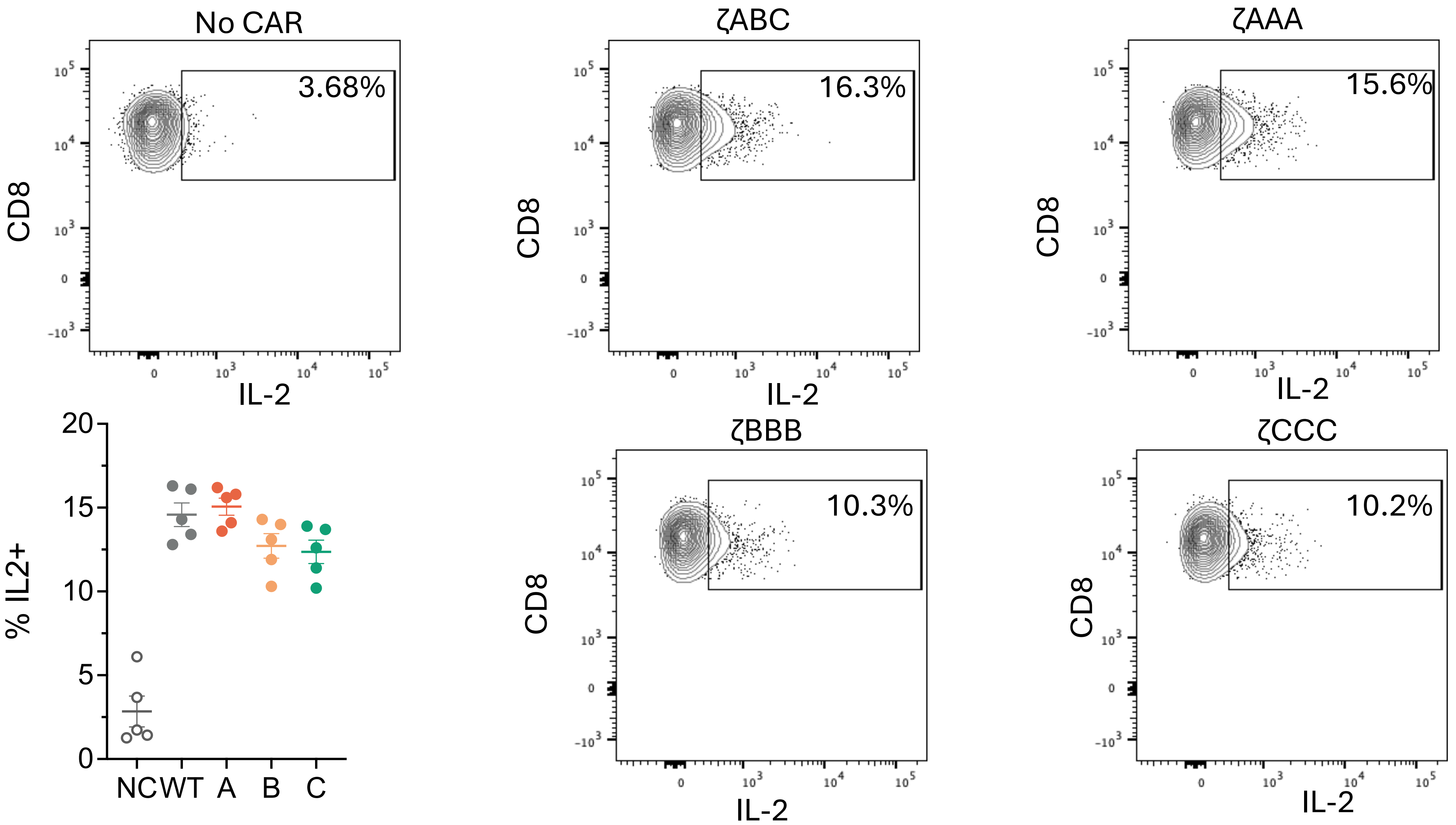

Supplemental Figure 2: In vitro functional characterization of ITAM restricted CAR-T cells suggest Zeta B and Zeta C CARs have weaker functional activation profile upon antigen encounter. IL-2 Cytokine profile of CD8+ CAR-T cells stimulated with CD19+ E-u cells in 1:2 E:T ratio for 6hrs in presence of BFA and monensin. Data pooled from 3 independent experiments.

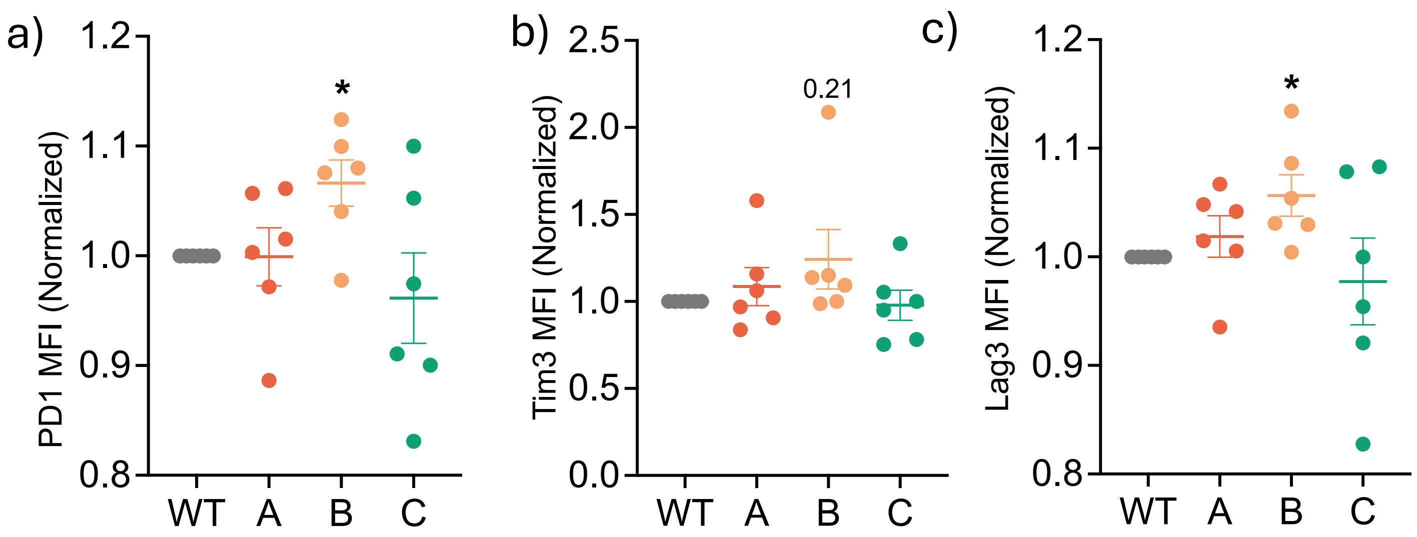

Supplemental Figure 3: Long-term in vitro stimulation of ITAM restricted CARs suggest Zeta B are more prone to exhaustion and Zeta C CARs are less prone to exhaustion. a-c) Normalized expression of PD1, Tim3 and Lag3 in CD8+ CAR-T cells at Day7 compared to WT. Data pooled from two independent experiments. Each dot represents an individual mouse donor.
